# Risk factors for Carbapenem-resistant *Acinetobacter baumannii* contamination on hospital surfaces: a multi-year environmental monitoring study in Shanghai, China

**DOI:** 10.3389/fmicb.2025.1609148

**Published:** 2025-06-19

**Authors:** Xing Zhang, Liang Tian, Jiaying Wu, Taiyao Chen, Tingting Shi, Yilin Ge, Renyi Zhu, Jian Chen

**Affiliations:** ^1^Department of Infectious Disease Control, Shanghai Municipal Center for Disease Control and Prevention, Shanghai, China; ^2^Department of Pathogen Biological Laboratory, Shanghai Municipal Center for Disease Control and Prevention, Shanghai, China

**Keywords:** carbapenem-resistant *Acinetobacter baumannii*, environment, risk factor, hospital, infection

## Abstract

**Background:**

Carbapenem-resistant *Acinetobacter baumannii* (CRAB) poses a significant threat to human health in hospital settings. These environments could serve as a reservoir for CRAB, since *Acinetobacter baumannii* (AB) exhibits strong survival capabilities outside the human body. Therefore, it is necessary to investigate the distribution of CRAB in the environment and identify the risk factors associated with its positive detection rate.

**Methods:**

From 2018 to 2023, long-term environmental monitoring of surfaces around CRAB patients was conducted across 16 hospitals in Shanghai. During each quarter, 48 environmental samples, along with information about the samples, were collected. Bacterial isolates were collected, and antimicrobial susceptibility testing (AST) was performed in accordance with the Clinical and Laboratory Standards Institute (CLSI) guidelines (2019–2021 edition). The CRAB detection rate in the environmental samples was compared across different variables. For nominal categorical variables, intergroup differences were analyzed using Pearson’s chi-squared (χ^2^) test. In addition, logistic regression analysis was employed to identify risk factors associated with CRAB-positive environmental samples.

**Results:**

A total of 10,268 samples were included in this study, among which 391 tested positive for CRAB. The overall CRAB positivity rate on environmental surfaces was 3.81%. Significant differences in positivity rates were observed across hospital levels, departments, sampling locations, and exposure frequencies (*p* < 0.05). Compared to Class B secondary hospitals, the following hospital classes showed significantly higher risks of CRAB detection: Class A secondary hospitals (*OR* = 13.34, 95%*CI*: 3.25–54.79, *p* < 0.001), Class B tertiary hospitals (*OR* = 20.63, 95%*CI*: 5.10–83.49, *p* < 0.001), and Class A tertiary hospitals (*OR* = 8.77, 95%*CI*: 2.14–35.87, *p* = 0.003). Compared to internal medicine departments, environmental surfaces in the following high-risk departments demonstrated higher rates of CRAB detection: surgical departments (*OR* = 1.93, 95%*CI*: 1.23–3.05, *p* = 0.005) and intensive care units (ICUs) (*OR* = 3.10, 95%*CI*: 2.19–4.39, *p* < 0.001). Additionally, surfaces located inside wards (*OR* = 1.834, 95%*CI*: 1.230 ~ 2.736, *p* = 0.003) and those with high-touch frequency (*OR* = 1.467, 95%*CI*: 1.134 ~ 1.898, *p* = 0.003) were identified as risk factors for the positive detection rate of CRAB in the environment.

**Conclusion:**

Class A secondary hospitals and Class B tertiary hospitals should prioritize infection control measures to prevent the dissemination of CRAB. Special attention should be given to high-risk areas, such as the surgical department and ICU, with enhanced disinfection of high-touch surfaces within patient wards.

## 1 Introduction

Antibiotic resistance has emerged as a critical global public health challenge, posing significant threats to human health. *Acinetobacter baumannii* (AB), a notorious nosocomial pathogen, is often linked to healthcare-associated infections (HAIs). Infections caused by AB are associated with severe clinical outcomes, including ventilator-associated pneumonia, bloodstream infections, urinary tract infections, and secondary meningitis, often resulting in prolonged hospitalization and elevated mortality rates. With the increasing use of antibiotics, carbapenem-resistant *Acinetobacter baumannii* (CRAB) has become one of the most problematic antimicrobial-resistant bacteria worldwide (Wang et al., 2024). These strains are often resistant to many other commonly used antibiotics (Ibrahim et al., 2021). CRAB infections present significant challenges in healthcare settings, given their resistance to antibiotics, high mortality rates, and substantial healthcare costs (Nasr, 2020).

Contaminated hospital environments and healthcare workers (HCWs) are increasingly recognized as critical reservoirs and vectors in the transmission and persistence of multidrug-resistant organisms (MDROs) (Fernando et al., 2017; Otter et al., 2013). AB exhibits prolonged survival in hospital environments, notably persisting on inanimate surfaces, which contributes to its role as a resilient nosocomial pathogen (Chapartegui-González et al., 2018). Studies have shown that AB can survive on dry inanimate objects for periods ranging from 3 days to as long as 11 months (Wagenvoort and Joosten, 2002). This prolonged environmental survival poses a significant risk for nosocomial transmission, facilitating the spread of HAIs. Previous research has shown that the risk of acquiring AB infection is 3.5 times higher in wards previously occupied by patients infected with AB (Chemaly et al., 2014). Although many studies have investigated common MDROs in hospitals (Lan et al., 2024; Nutman et al., 2020; Nutman et al., 2023), the role of the environment in transmitting MDROs remains underestimated (Chemaly et al., 2014). There is a growing link between a contaminated environment in medical institutions and the persistence of MDROs (Fernando et al., 2017). Healthcare settings were identified as a risk factor for patients developing antimicrobial-resistant co-infections during the COVID-19 pandemic (Kariyawasam et al., 2022). Environmental disinfection is one of the important measures to address antimicrobial-resistant problems (Anderson et al., 2017; Chemaly et al., 2014). Therefore, it is necessary to identify the areas of severe antibiotic-resistant bacterial contamination in hospital settings, which can provide a basis for targeted environmental disinfection and improve disinfection efficiency.

In this study, we characterized the distributional features of CRAB in the hospital environment. Furthermore, we explored the risk factors associated with the positive detection of CRAB in these settings.

## 2 Methods

### 2.1 Environmental sampling

We conducted longitudinal environmental surveillance of CRAB in inpatient settings across Shanghai. Using a convenience sampling approach, one hospital was selected from each of Shanghai’s 16 administrative districts. From the second half of 2018 to the first half of 2023, surface samples were collected quarterly from high-touch areas in critical departments (e.g., intensive care units (ICUs) and surgical wards). Sampling was performed during routine clinical care without restricting collection to pre- or post-disinfection periods to reflect real-world microbial colonization. Specimens were obtained from environmental surfaces proximal to CRAB-infected patients (e.g., bed rails and infusion pumps). Concurrently, metadata including sampling location, time, and patient contact frequency were recorded.

### 2.2 Strain collection

The BD 100 system and the VITEK MS automatic rapid microbial mass spectrometry system were used to identify the bacteria. Quality control strains included *Escherichia coli* (ATCC 25922) and *Pseudomonas aeruginosa* (ATCC 27853).

### 2.3 Antimicrobial drug sensitivity testing

Antimicrobial susceptibility testing (AST) was performed. The microbroth dilution method was used to determine the minimum inhibitory concentration (MIC) of antibacterial agents. Sensitivity (S), intermediate (I), and resistance (R) results were obtained according to the 2019–2021 version of the American Society for Clinical Laboratory Science (CLSI) guidelines.

### 2.4 Definitions

According to China’s Hospital Classification Management Standards, public hospitals in China are divided into three levels, each further subdivided into grades. Primary hospitals are community-based healthcare centers that deliver preventive care, medical treatment, health maintenance, and rehabilitation services to a defined population. Secondary hospitals serve as regional medical hubs, offering comprehensive healthcare services across multiple communities while also undertaking teaching and research responsibilities. Tertiary hospitals operate at a supra-regional level, providing advanced specialized medical services and engaging in higher education and research across broader regions. In addition, based on the evaluation scores from Part III of the Criteria for Accreditation of the Three Levels, hospitals are classified into three grades: Grade A (requiring a score of at least 90%), Grade B (≥80%), and Grade C (≥70%). Given that this study primarily examined hospital environments for inpatients—most of whom receive care in secondary and tertiary hospitals—the research was conducted within these two tiers. Specifically, the study encompassed the following hospital classes, listed in order from lower to higher levels: Class B secondary hospitals, Class A secondary hospitals, Class B tertiary hospitals, and Class A tertiary hospitals.

The frequency of exposure was classified as low, middle, and high based on the actual contact frequency of patients during daily care and previous studies (Cheng et al., 2015). Low-touch frequency surfaces included electrical switches, kicks, therapy trolleys, office cabinets, and telephones. Middle-touch frequency surfaces included IV stands, showerheads, garbage bins, infusion pumps, and doorknobs. High-touch frequency surfaces included bed rails, handles, grab bars, toilet seats, beepers, bedside cabinets, and sphygmomanometers.

### 2.5 Statistical analysis

The detection rate of CRAB in the environmental samples was compared across different variables. Pearson’s chi-squared (χ^2^) test was used for nominal categorical variables. To identify risk factors associated with CRAB-positive environmental samples, a multivariable logistic regression analysis was conducted, incorporating the following variables: year, hospital level, department, sample location, and frequency of exposure. A two-sided *p*-value < 0.05 was considered statistically significant. All analyses were performed using SPSS (version 25.0).

## 3 Results

### 3.1 Baseline

Due to the COVID-19 pandemic, most hospitals implemented full closures during the initial phase of the outbreak, leading to an interruption of this study in 2020. As the pandemic’s impact varied across hospitals, the timing of research resumption differed by institution. From 2018 to 2023, a total of 13,281 environmental samples were collected. After excluding fabric-related surface samples, 10,268 samples were retained for analysis. Of these, 391 samples tested positive for CRAB, yielding an average environmental surface contamination rate of 3.81%.

### 3.2 The detection rate of CRAB varied across different variables

As shown in Table 1, from 2018 to 2023, the positive rates were 3.01, 3.24, 5.43, 3.66, 3.83, and 3.68%, respectively (*χ^2^* = 16.489, *p* = 0.006). There were statistically significant differences based on hospital level, department, sample location, and frequency of exposure. The positive detection rates of CRAB in Class B secondary hospitals, Class A secondary hospitals, Class B tertiary hospitals, and Class A tertiary hospitals were 0.38, 2.94, 6.09, and 2.38%, respectively (*χ^2^* = 95.382, *p* < 0.001). The positive detection rate was 1.52% in the internal medicine department, which was lower than that in the ICU (4.87%) and surgical department (3.37%) (*χ^2^* = 58.647, *p* < 0.001). The positive detection rate was higher in the samples collected from inside the wards (4.20%) than in those collected from outside the wards (1.86%) (*χ^2^* = 21.548, *p* < 0.001). Regarding frequency of exposure, the positive detection rates were 3.34, 3.54, and 4.98% for low-, middle-, and high-touch frequency surfaces, respectively (*χ^2^* = 12.735, *p* = 0.002).

**Table 1:** Distribution of CRAB positive detection rates across different variables.

Variables	Positive number of samples	Total number of samples	Positive rate
Year			
2018	34	1,131	3.01
2019	77	2,379	3.24
2020	92	1,694	5.43
2021	90	2,459	3.66
2022	52	1,356	3.83
2023	46	1,249	3.68
Hospital level			
Class B secondary hospital	2	525	0.38
Class A secondary hospital	77	2,621	2.94
Class B tertiary hospital	234	3,840	6.09
Class A tertiary hospital	78	3,282	2.38
Department			
ICU	308	6,325	4.87
Internal medicine department	41	2,697	1.52
Surgical department	42	1,246	3.37
Sample location			
Inside of wards	359	8,544	4.20
Outside of wards	32	1,724	1.86
Frequency of exposure			
Low	149	4,461	3.34
Middle	116	3,275	3.54
High	126	2,532	4.98

### 3.3 Risk factors for positive CRAB detection rates in the hospital environment

As shown in Table 2, there was no statistically significant difference in CRAB detection rates between years. Compared to class B secondary hospital, Class A secondary hospital (*OR* = 13.338,95%*CI*: 3.247 ~ 54.790, *p* < 0.001), Class B tertiary hospital (*OR* = 20.629, 95%*CI*:5.097 ~ 83.492, *p* < 0.001), and Class A tertiary hospital (*OR* = 8.767, 95%*CI*:2.143 ~ 35.874, *p* = 0.003) had a higher risk of positive CRAB detection. Compared to internal medicine departments, surgical departments (*OR* = 1.934, 95%*CI*: 1.226 ~ 3.051, *p* = 0.005) and ICUs (*OR* = 3.104, 95%*CI*: 2.193 ~ 4.392, *p* < 0.001) had a higher risk of positive CRAB detection on environmental surfaces. Compared to environmental surfaces outside wards, environmental surfaces inside wards (*OR* = 1.834, 95%*CI*: 1.230 ~ 2.736, *p* = 0.003) had a higher risk of CRAB detection. Compared to environmental surfaces with low-frequency patient contact, environmental surfaces with high-frequency patient contact (*OR* = 1.467, 95%*CI*: 1.134 ~ 1.898, *p* = 0.003) had a higher risk of CRAB detection (Table 2).

**Table 2:** Risk factors identified by logistic regression analysis.

Variables	b	Sb	*Wald χ^2^*	*p*-value	*OR*	*95%CI*
Year						
2018					1.000	
2019	−0.062	0.215	0.083	0.773	0.940	0.616 ~ 1.433
2020	0.369	0.213	3.002	0.083	1.446	0.953 ~ 2.193
2021	−0.083	0.213	0.150	0.698	0.921	0.606 ~ 1.398
2022	−0.058	0.233	0.062	0.804	0.944	0.598 ~ 1.490
2023	−0.106	0.238	0.201	0.654	0.899	0.564 ~ 1.432
Department						
Internal medicine department					1.000	
Surgical department	0.660	0.233	8.043	**0.005**	**1.934**	1.226 ~ 3.051
ICU	1.133	0.177	40.870	**<0.001**	**3.104**	2.193 ~ 4.392
Hospital level						
Class B secondary hospital					1.000	
Class A secondary hospital	2.591	0.721	12.914	**<0.001**	**13.338**	3.247 ~ 54.790
Class B tertiary hospital	3.027	0.713	18.004	**<0.001**	**20.629**	5.097 ~ 83.492
Class A tertiary hospital	2.171	0.719	9.120	**0.003**	**8.767**	2.143 ~ 35.874
Sample location						
Outside wards					1.000	
Inside wards	0.606	0.204	8.838	**0.003**	**1.834**	1.230 ~ 2.736
Frequency of exposure						
Low					1.000	
Middle	−0.096	0.131	0.534	0.465	0.908	0.702 ~ 1.175
High	0.383	0.131	8.527	**0.003**	**1.467**	1.134 ~ 1.898

*The bold values represented the *p*-value, which was <0.05.

## 4 Discussion

We conducted a longitudinal surveillance study across 16 hospitals in Shanghai to assess CRAB contamination on environmental surfaces in healthcare facilities and to characterize its distribution patterns. In addition, we quantified potential risk factors associated with CRAB contamination. During the 5-year study period (2018–2023), the mean CRAB detection rate across all sampled environmental surfaces was 3.81% (391/10268). The results indicated that the risk of positive CRAB detection on environmental surfaces was relatively higher in Class B tertiary hospitals, ICUs, inside wards, and on surfaces with a high frequency of exposure. These areas should be given more attention during routine infection prevention and control efforts.

Our findings demonstrated that the detection rate of CRAB varied significantly across different years, hospital levels, hospital departments, sampling locations, and exposure frequencies. The detection rate of CRAB varied significantly across different years (*p* = 0.006). However, multivariate logistic regression analysis revealed that the year of sampling was not a risk factor for CRAB contamination in the hospital environment. Notably, the CRAB detection rate peaked in 2020 (5.43%) compared to other study years (range: 3.01–3.83%), potentially reflecting COVID-19 pandemic-related impacts on infection control measures. Antimicrobial resistance (AMR) has been a hidden threat lurking behind the COVID-19 pandemic (Rizvi and Ahammad, 2022). The COVID-19 pandemic may have contributed to an increased prevalence of antimicrobial resistance (AMR), particularly among Gram-negative pathogens (Langford et al., 2023). Although it was indicated that the COVID-19 pandemic could improve infection prevention and control practices globally (Collignon and Beggs, 2020), a shortage of medical personnel and personal protective equipment during the pandemic may have facilitated the transmission of AMR (Langford et al., 2023), especially in areas affected by poverty and weak regulatory frameworks. This may explain why we observed increased detection rates in 2020.

The prevalence of CRAB also differed across different levels of hospitals. As observed in the results, hospital level is a risk factor for the positive detection rate of CRAB in the hospital environment. Consistent with China’s Hospital Classification Management Standards, tertiary hospitals typically maintain advanced infection prevention and control (IPC) systems (Tao et al., 2024) and have greater bed capacity, comprehensive infrastructure, and superior technical and managerial capabilities compared to lower-level healthcare facilities. In this study, Class B tertiary hospitals had higher CRAB detection rates (6.09%, 234/3840), which may be due to their larger bed capacity and relatively weaker technical and managerial capabilities compared to Class A tertiary hospitals (2.38%, 78/3282). In contrast, Class B secondary hospitals, with fewer inpatients and a smaller proportion of severely ill patients with weakened immune function, tended to have relatively lower detection rates of CRAB (0.38%, 2/525). The findings indicate that Class A secondary, Class B tertiary, and Class A tertiary hospitals should place greater emphasis on infection control and prevention of CRAB dissemination.

It was revealed in this study that surgical departments and ICUs had a higher risk of positive CRAB detection on environmental surfaces compared to internal medicine departments. Surgical departments are particularly vulnerable to HAIs and antimicrobial-resistant pathogens due to the frequent use of invasive diagnostic and therapeutic procedures that compromise natural barriers to infection. It has been shown that recent surgery and invasive interventions are significant independent predictors of AB infection in patients with pneumonia (Lan et al., 2024). Patients in the intensive care unit are usually in critical condition, have a low level of immune function, and are undergoing invasive procedures (Jung-Jr et al., 2010). Therefore, the intensive care unit is a priority area for the prevention and control of drug-resistant bacteria. The elevated CRAB contamination frequency observed on ICU surfaces may partially reflect selection bias in our sampling strategy. As active surveillance cultures were routinely obtained from ICU patients but not from general ward patients, environmental sampling density was consequently higher in critical care areas, potentially increasing the likelihood of detecting CRAB.

Surfaces of objects around patients have a higher risk of contamination than other sites (Otter et al., 2013). As the primary source of CRAB transmission, patients mainly reside and move within wards. Therefore, in this study, the detection rate of CRAB on environmental object surfaces inside the wards was higher compared to samples collected outside the wards. High-touch surfaces are recognized as a potential reservoir of infectious pathogens, and their contamination could also pose a significant threat to the dissemination of multi-resistant organisms (Casini et al., 2018; Li et al., 2021). In medical institutions in China, single-occupancy wards are relatively rare, with most wards being multi-occupancy. Notably, the shortage of single rooms hinders the effective isolation of patients known to be colonized or infected with pathogens (Otter et al., 2013). Therefore, the risk of cross-infection of CRAB within multi-patient wards is a significant concern. In addition, as conventional cleaning may not always reach all environmental reservoirs (Robustillo-Rodela et al., 2017), terminal cleaning and disinfection may not reliably eliminate pathogens. Enhancing environmental disinfection measures may be effective in removing CRAB and reducing HAIs (Donskey, 2013). Overall, in the management of patients infected with CRAB, special attention should be given to environmental surfaces within the wards. It is essential to strengthen environmental disinfection in high-risk departments with a higher likelihood of detecting CRAB, as well as on object surfaces that are frequently touched by patients.

Our study explains the distribution of CRAB in the environment and explores the risk factors associated with its positive detection rate. However, there are still some limitations to this study. First of all, due to logistical constraints associated with on-site sampling in clinical environments, this study employed a convenience sampling approach. Although this method ensured feasibility, it might have limited the generalizability of our findings to broader hospital settings. Secondly, 3,013 fabric-related samples were excluded from this study since this research mainly focused on surfaces of ordinary objects in the environment. However, the clothing of healthcare workers could play a role in the transmission of microorganisms (Chemaly et al., 2014). In addition, AB has been isolated from washing machines and textiles (Bockmühl, 2017), suggesting that these could serve as important sources of CRAB transmission via direct body contact during daily care. The role of textiles in transmitting CRAB during daily healthcare activities could be investigated in future studies. Thirdly, the mechanisms underlying the transmission of CRAB between the environment and patients have not yet been thoroughly explored. In most Chinese public hospitals, patient rooms are typically multi-occupancy wards, which makes infection prevention and control more challenging. Therefore, to interrupt CRAB transmission in the hospital environment, it is important to study the mechanisms underlying CRAB transmission between the environment and patients. This understanding could serve as the foundation for developing strategies to prevent the spread of CRAB in multi-occupancy wards.

## 5 Conclusion

This longitudinal surveillance study provides critical insights into the epidemiological distribution of CRAB within hospital environments. It is necessary for hospitals to maintain an adequate supply of personal protective equipment, notably during the outbreak of epidemics. It is essential for Class A secondary hospitals, Class B tertiary hospitals, and Class A tertiary hospitals to prioritize healthcare-associated infection control and the prevention of CRAB dissemination. Special attention should be given to environments of surgical departments and ICUs, especially the surfaces located inside wards and those with high-touch frequency, during routine disinfection practices.

## Data Availability

The original contributions presented in the study are included in the article/supplementary material, further inquiries can be directed to the corresponding authors.
